# The impact of participation in agricultural industry organizational models on crop yields: evidence from Chinese wheat growers

**DOI:** 10.1038/s41598-023-43879-0

**Published:** 2023-10-18

**Authors:** Xinde Zhang, Xiaoyu Wan, Pengling Liu

**Affiliations:** 1 School of Economics and Management, Anhui Agricultural University, Hefei, China; 2 School of Mathematics and Statistics of Hefei Normal University, Hefei, China; 3 Anhui Agricultural University, Hefei, China

**Keywords:** Socioeconomic scenarios, Sustainability

## Abstract

Increasing income for grain farmers is crucial to mobilise farmers’ incentive for grain cultivation. This article, based on the survey data of 2032 wheat growers in Anhui Province, China, employs the Logit model, multinomial Logit model, and entropy balance-OLS regression method to empirically analyze the factors influencing wheat growers’ participation in agricultural industrial organization models and the impact of their participation decisions on planting returns. The research found that: (1) Wheat growers with richer resource endowments are more likely to participate in agricultural industrial organization models. Factors such as household head’s education level, training experience, quality of arable land, scale of operation, and labor endowment are crucial determinants of wheat growers’ decisions to participate in industrial organization models. (2) Participation in agricultural industrial organizations significantly enhances the net income of wheat growers. Comparatively, the income-boosting effect is more pronounced for those participating in the “household + farmer cooperatives/agricultural enterprises” model. (3) The mechanisms through which wheat growers’ participation in different agricultural industrial organization models affects their crop yields vary. The income-enhancing effects of wheat growers' participation in the “household + farmer cooperatives/agricultural enterprises” model of industrial organization primarily stem from the improvement in land productivity and market bargaining power. On the other hand, the income-enhancing effects of participation in the “household + village collective + farmer cooperatives/agricultural enterprises” model are mainly attributed to the improvement in market bargaining power. The policy implication is that priority should be given to cultivating and developing industrial organisations based on the model of “household + farmers’ co-operatives/agribusinesses” in regions where farmers are richly endowed with resources, and at the same time, the development of industrial organisations based on the model of “household + village collectives + farmers’ co-operatives/agribusinesses” should be supplemented in accordance with local conditions. At the same time, the development of “household + village collectives + farmers’ cooperatives/agribusinesses” mode is supplemented according to local conditions.

## Introduction

As a transition country with a large global population, China's stable food supply holds significant importance in balancing global food trade and alleviating global poverty^1^.

Since the 18th National Congress of the Communist Party of China, the central government has consistently prioritized food security as a top priority in the governance. It has established the Chinese characteristic new grain security strategy, emphasizing “self-reliance, domestic focus, capacity assurance, moderate imports, and technological support”. This strategy underscores the concept of “ensuring basic self-sufficiency in grains and absolute food security”.

At the 20th Party Congress of the CPC, President Xi Jinping of the People’s Republic of China emphasized that “Comprehensively consolidate the foundation of food security…Ensure that the Chinese people’s rice bowls are firmly in their hands”. In 2023, the Central Document No. 1 identified “making every effort to improve grain production” as the top priority for the year.

**Figure 1:**
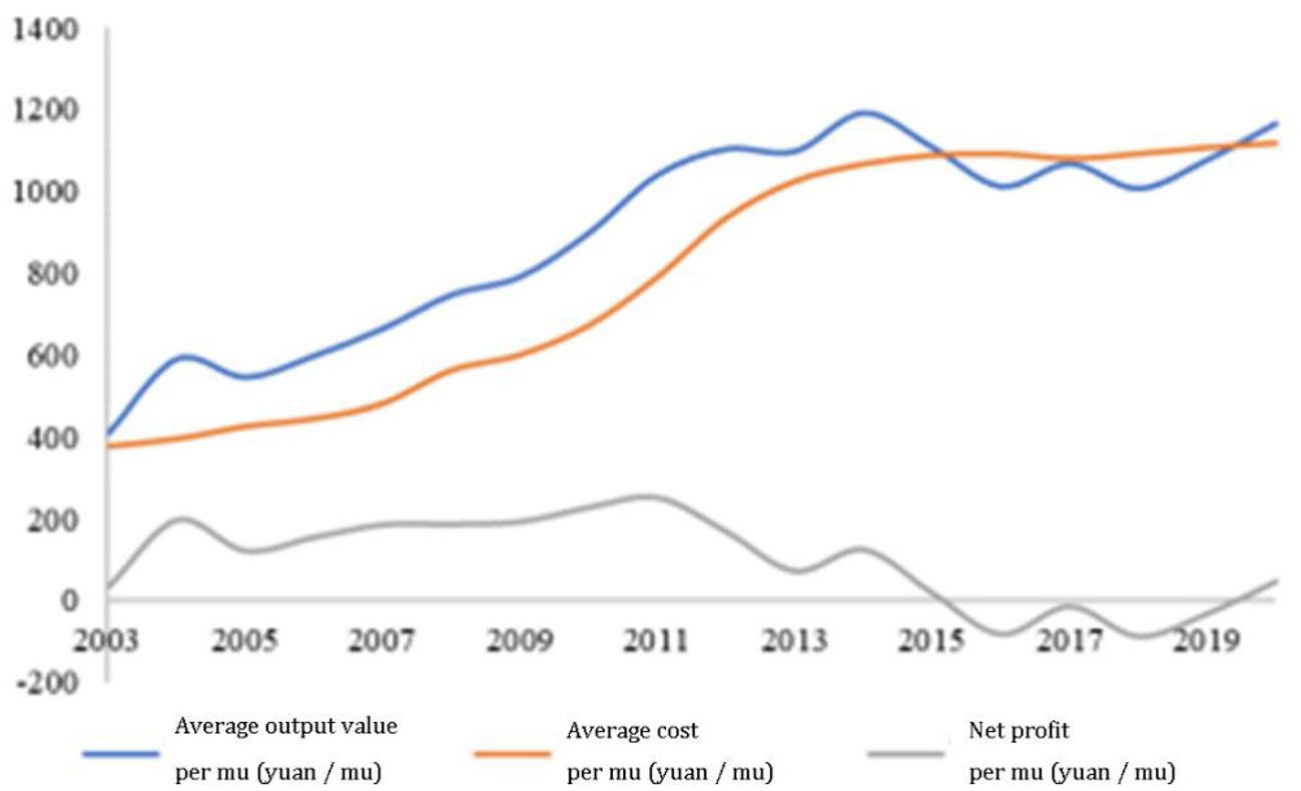
Cost–benefit comparison of three major grain crops in China (2003–2020).

However, under the national and agricultural conditions of more people and less land, there is a significant inherent contradiction between ensuring the absolute safety of grain rations and increasing the farmer's income. On the one hand, China has established a system of provincial governors assuming responsibility for "grain production" and the assessment mechanism based on "food security party and government responsibility", in order to enhance the local government's consciousness of focusing on agriculture and grain, and to guide more labor and land resource to be allocated to grain production. On the other hand, due to the rapid rise in grain production costs and prices, and the rigid rise in farmland rent, the comparative income of grain planting is low. According to the *Summary of National Agricultural Product Cost–Benefit Data* in 2021 (Fig. 1), the net profit per-mu of China’s three primary grains (rice, wheat, corn) has decreased dramatically from 2011. It has even been negative from 2016 to 2019. Furthermore, due to the impact of COVID-19, the international trade situation and the Russia-Ukraine conflict, global food markets volatility may increase further^2,3^, and China’s domestic grain supply and demand will constantly be in a tight balance^4^. Faced with the uncertain impact of the factors mentioned above, how to realize the coordination between the absolute safety of grain rations and the increase of farmers' income to ensure agricultural productivity and enhance grain production capacity is an important issue that requires exploration urgently.

To alleviate the above contradictions, the Chinese government has issued a series of policy measures since the 1990s to support and encourage new agricultural business entities, such as agricultural leading enterprises and farmer cooperatives, promote agricultural industrial organization mode innovation, and change the independent production and management mode of scattered smallholder farmer to promote the cost-saving and income-increasing of grain planting entities. The academics have studied the innovation of industrial organization mode, solving the problems of low degree of agricultural production organization, low agricultural competitiveness, and difficulty in increasing smallholder farmer' income.

Researchers have explored the meaning and types of industrial organization models^5^, factors influencing the participation of household in industrial organization models, and the beneficial effects of farmers’ involvement in different industrial organization models^6^. However, existing research has primarily focused on single organizational models such as agricultural leading enterprises + household or farmer cooperatives + household. This narrow focus has overlooked the diversity of agricultural industrial organization models in China and the differences in welfare effects on farmers among various organizational models. Furthermore, previous research has placed emphasis on cash crops like vegetables and fruits, as well as the livestock industry, with limited attention given to the development of grain industry organizational models and their impact on the income of grain farmers. There is a pressing need for research in this area.

This paper focuses on the wheat industry in Anhui Province, China's central province of net grain export. Based on a comprehensive understanding of grain industrial organization types, this paper uses the questionnaire survey data of wheat growers to analyze the influencing factors of grain growers' participation in different industrial organization modes. It empirically examines the impact and mechanism of different industrial organization modes on grain growers' income. Based on the participants and operating mechanisms of different industrial organization models, this paper divides this model in the grain field into three categories: "household," "household + farmer cooperatives/agricultural enterprises," and "household + village collective + farmer cooperatives/agricultural enterprises.", and focuses on the householder, family and regional characteristics of wheat growers. Logit and multinomial Logit models are used to investigate the influencing factors of wheat growers' participation in different industrial organization models. Subsequently, the Entry Balancing data preprocessing method proposed by Hainmueller was utilized to investigate the impact and mechanism of different industrial organization models on the planting income of wheat growers based on alleviating the sample selection bias^7^.

## Data description and model setting

### Data source

The data used in this paper are from a questionnaire survey of wheat growers in Anhui Province. Anhui Province is one of China’s five net grain exporting provinces. The province’s grain planting area reached 7309.6 thousand hectares in 2021, ranking fourth among China’s 34 provincial administrative regions. Wheat is the most important grain crop in Anhui Province. In 2021, the wheat planting area in the province was 2846 thousand hectares, accounting for 42.97% of the total grain planting area, distributed in 15 cities except Huangshan City. In 2021, wheat output in the province reached 16.9973 million tons, accounting for 42.77% of the total grain output. This survey began in August 2022 and was divided into two stages. The first stage is to select Lujiang County and Changfeng County in Anhui Province for a questionnaire survey and in-depth interview. The subjects of the survey and interviews included wheat growers, government staff, farmers’ cooperatives, agricultural enterprises and other subjects, with the aim of understanding the basic types and operation mechanisms of industrial organizations in wheat growing in Anhui Province, and further improve the research questionnaire on this basis, the final questionnaire included basic information of farmers, farmers' participation in agricultural industry organizations, wheat planting inputs and income, etc. The second stage uses a stratified random sampling method to determine the sample collection point (specific to the township) and conduct a formal questionnaire survey. Firstly, according to the median wheat planting area in 15 prefecture-level cities under the jurisdiction of the country (cities, districts) were divided into two groups: large wheat-growing counties (cities, districts) and small wheat-planting counties (cities, districts), and 15 counties (cities, districts) were randomly selected in each of the two groups, totaling 30 counties (cities, districts). Subsequently, 1–2 townships (towns) were randomly selected from each sample county as a questionnaire collection point for this survey. The final selected research sites cover 41 townships in Anhui Province. Finally, wheat growers were randomly selected according to the list of wheat growers provided by the sample township (town) agricultural authorities, and a questionnaire survey was conducted through a one-to-one questionnaire survey. A total of 2050 questionnaires were distributed in this survey, and finally, 2032 valid questionnaires were obtained from wheat growers, with an effective rate of 99.17%.

### Typical fact analysis

This article draws upon the definitions and classification criteria of agricultural industrial organization models^8^. It classifies the types of agricultural industrial organization models based on the classification of the operating entities involved in these models. Through in-depth interviews and questionnaire surveys, it was found that the agricultural industrial organization models in the sampled regions exhibit diverse characteristics, including eight kinds of industrial organization models, respectively, "household," "household + cooperative," "household + enterprise," "household + cooperative + enterprise," "household + village collective," "household + cooperative + village collective," "household + village collective + enterprise," and "household + cooperative + village collective + enterprise.". As shown in Table 1, wheat growers in the sampled regions have a relatively high degree of organization. In the survey year, 58.15% of the sampled farmers participated in at least one agricultural industrial organization model, while 40.85% operated independently as household. Among the organizational models, the "household + farmer cooperative" model had the highest participation rate. This may be attributed to the enactment of China’s first "Farmers' Specialized Cooperative Law" in 2007 and the introduction of a series of supportive policies, which accelerated the development of farmer cooperatives in China. By the end of 2022, there were 2.2436 million active farmer cooperatives in China, benefiting nearly half of the farmers. Additionally, village collectives play a significant role in reshaping agricultural industrial organization models. The survey found that 23.09% of household joined industrial organizations with village collectives involved. This not only helps reduce transaction costs for household when entering contracts with farmer cooperatives and agricultural enterprises but also mitigates issues related to the bargaining power of household.

**Table 1:** Types of industrial organization models for wheat planting farmers.

Industrial organization model	Number	Proportion	Industrial organization model	Number	Proportion
Household	830	40.85	Household + cooperatives + enterprises	107	5.27
Household + enterprises	23	1.13	Household + cooperatives + village collectives + enterprises	272	13.39
Household + village collectives + enterprises	26	1.28	Household + village collectives	40	1.97
Household + village cooperatives	603	29.68	Household + village collectives + enterprise	131	6.45

In this study, wheat growers are initially divided into two categories: the treatment group and the control group based on whether they participate in agricultural industrial organizations. Specifically, wheat growers who participate in any industrial organization model are categorized as the treatment group, while those who do not participate in any industrial organization model are considered the control group.

The cooperative model often operates at the household level, utilizing the resources of individual households to engage in collaborative production. By helping each other, driving growth, benefiting from policy support, and collectively expanding production scale in a specific field, the cooperative model aims to achieve economies of scale. Firstly, when purchasing production materials or organizing the sale of agricultural products, this model can leverage economies of scale, thereby reducing the operational costs for individual farmers. Additionally, through collective decision-making and access to comprehensive market information, it can lower farmers' business risks, prevent blind operations, and to some extent, mitigate the impact of market competition. Secondly, cooperative approaches can increase influence, enhance rural residents' enthusiasm for entrepreneurial activities, and expedite the development of projects into industrialization and scale in a region. This model is conducive to achieving industrial, product, and regional scale within a specific area, facilitating large-scale operations. The company cooperative model is also a widely explored approach by farmers. In this model, farmers contribute land or capital to establish a company as shareholders. Under this structure, the company manages operations, addressing production disparities arising from varying individual capabilities among cooperative members. Companies maintain separate accounting, shareholders share profits and risks, and farmers can directly participate in production. With unified pricing and market sales under the company model, there is no issue of revenue loss due to competition among partners in the same market, significantly boosting farmers' motivation and promoting regional industrialization. Village collectives refer to community-based rural collective economic organizations that operate under the dual-layer rural management system. They are collectively owned, cooperatively operated, democratically managed, and serve their members. Village collectives are supervised by township governments and work in coordination with village committees. They allocate necessary funds to fulfill village-level organization functions and adequately manage public affairs and public welfare projects in the village.

Considering that there is no significant difference between farmers' cooperatives and agribusinesses in China and the mechanisms linking farmers with farmer cooperatives or agricultural enterprises are relatively loose, village collective economic organizations in many regions participate in the reshaping of industrial organization models. Additionally, there is limited research data on some of these models. Therefore, based on the types of agricultural industrial organization models in which wheat growers participate, this study categorizes wheat growers who have not yet participated in any industrial organization as the control group (referred to as "smallholder farmers farmers" hereafter). The treatment group is further divided into two main categories: "household + agricultural enterprise/farmer cooperative" (Treatment Group I) and "household + village collective + cooperative/farmer enterprise" (Treatment Group II), as shown in Table 2.

**Table 2:** The categorization of wheat growers based on the types of industrial organization models.

Group	Types of industrial organization models
Control group	Household (wheat growers who have not yet participated in any industrial organization)
Treatment group I	Household + enterprise
	Household + farmer cooperative
	Household + farmer cooperative + enterprise
Treatment group II	Household + agricultural enterprise + village collective
	Household + farmer cooperative + enterprise + village collective
	Household + village collective
	Household + village collective + farmer cooperative

Table 3 presents data on crop yields, input costs, and wheat selling prices for different categories of wheat growers. Mean t-tests reveal noteworthy findings: wheat growers participating in industrial organization models experience a substantial reduction in physical costs for wheat cultivation (including expenses such as pesticides and fertilizers) and mechanical input costs, compared to those who have not yet engaged in any industrial organization. Moreover, Treatment Group I demonstrates a certain degree of improvement in per-hectare yield. Table 3 also highlights that wheat growers engaged in industrial organization models command higher selling prices for their wheat compared to those who have not participated in such models. Additionally, wheat growers participating in models involving village collectives exhibit stronger negotiation capabilities in the market. As illustrated in Table 3, participation in industrial organization models tends to lead to enhanced net crop yields for wheat growers.

**Table 3:** Means T-test for outcome variables.

	Control group (a)	Treatment group (b)	Treatment group I (c)	Treatment group II (d)	(a)–(b)	(a)–(c)	(a)–(d)
Revenue per mu (1000CNY)	0.133 (0.112)	0.127 (0.110)	0.129 (0.120)	0.124 (0.086)	0.006 [1.19]	0.004 [0.672]	0.009 [1.553]
Physical cost per mu (CNY)	323.986 (237.210)	306.508 (271.964)	302.679 (264.209)	301.873 (265.662)	18.881 [1.550]	17.478 [1.299]	21.308 [1.387]
Machinery cost per mu (CNY)	100.035 (101.205)	94.343 (128.899)	95.505 (120.518)	92.319 (142.468)	5.692 [1.022]	4.530 [0.782]	7.717 [1.071]
Yield per mu (500g)	879.500 (346.211)	872.381 (354.723)	881.458 (350.285)	856.688 (362.137)	7.120 [0.446]	− 1.958 [− 0.111]	22.812 [1.093]
Wheat price (CNY/500g)	1.333 (0.147)	1.359 (0.146)	1.359 (0.146)	1.367 (0.156)	− 0.029*** [− 4.170]	− 0.027*** [− 3.503]	− 0.032*** [− 3.486]

Values in parentheses are standard deviations and values in square brackets are t-values.

If P < 0.05, the significance is * ; If P < 0.01, the significance is **; If P < 0.001, the significance is ***.

### Measurement model setup

#### Decision-making model for industrial organization model selection

The industrial organization choice decision of wheat growers is a two-dimensional choice decision. Therefore, this paper uses Logit model to examine the influencing factors of their choice decision empirically. The basic form of the model is as follows:

1 $$P(Optio{n}_{i}=1\left|{x}_{i})\right.=F({x}_{i},\beta )=\Lambda ({x}_{i}{\prime}\beta )\equiv \frac{\mathit{exp}({x}_{i}{\prime}\beta )}{1+\mathit{exp}({x}_{i}{\prime}\beta )}.$$

Among them, *i* represents the wheat growers, and $Optio{n}_{i}$ refers to choice decision for the industrial organization model of the wheat growers *i* . If wheat growers *i* choose to participate in any industrial organization model, then $Optio{n}_{i}=1$, otherwise it is 0. *F*(∙) is the cumulative distribution function of the logical distribution, *x*_i_ is a set of exogenous variables that affect the choice decision for the industrial organization model of the wheat growers. This paper selects the personal characteristics of householder^9–11^, the characteristics of farmer households and the terrain conditions of the village (Table 4). β is the corresponding estimated parameter. In addition, considering that the economic meaning of the estimated coefficients of the Logit model is difficult to explain, this paper uses the Linear Probability Model (LPM) to estimate whether farmers participate in industrial organization as the dependent variable.

**Table 4:** Control variables: definition and descriptive statistics.

Variable	Definition	Obs	Mean	Std. dev
Age of householder	Age of householder (years)	2032	47.0378	8.8885
Gender of householder	Male = 1, female = 0	2032	0.9218	0.2686
Education level of householder	The education level of householder is high school and above = 1, other = 0	2032	0.6319	0.4824
Householder training experience	There = 1, no = 0	2032	0.9286	0.2574
Cadre family marriage	Whether the interviewee's family is a cadre family marriage: yes = 1, no = 0	2032	0.3051	0.4605
Farmland confirmation	Completed = 1, unfinished = 0	2032	0.8991	0.3012
Cultivated land quality	Better or excellent = 1, other = 0	2032	0.2800	0.4491
Farmland transferred in	Yes = 1, No = 0	2032	0.7805	0.4140
Size of family members	People	2031	4.9389	1.9587
The scale of wheat operation	Mu (1 mu = 0.067 hectare)	1972	330.3786	570.8557
Proportion of migrant workers	The proportion of migrant workers in the number of family members (%)	2009	15.1956	19.6620
Labour share	The proportion of working-age population in the number of family members (%)	2016	61.7093	20.6368
Irrigation conditions	The proportion of irrigated arable land (%)	1958	75.2956	32.6174
Plain	Whether the area is a plain : yes = 1, no = 0	2032	0.7908	0.4068

To further reveal the decision-making mechanism of wheat growers participating in different industrial organization modes (smallholder farmer, smallholder farmer + cooperatives/agricultural enterprises, smallholder farmer + village collectives + cooperatives/agricultural enterprises), this paper assumes that the random utility of wheat growers *i* choosing industrial organization mode *j* is:

2 $${U}_{ij}={x}_{i}{\prime}{\beta }_{j}+{\varepsilon }_{ij} \left(i=1,\cdots ,n;j=\mathrm{1,2},3\right).$$

Among them, *x*_*i*_ has the same meaning with formula (1), it is a set of exogenous variables that affect the choice decision for the industrial organization model of the wheat growers, and *x*_*i*_ only changes with individual *i*, not with the type of industrial organization mode *j*. Obviously, if and only if the utility of industrial organization mode *j* is higher than that of all other industrial organization modes, wheat grower *i* chooses industrial organization mode *j*. Therefore, the probability of wheat grower *i* choosing industrial organization mode *j* can be set as:

$$P\left({Option}_{ij}=j|{x}_{i}\right)=P\left({U}_{ij}\ge {U}_{ik}, \forall k\ne j\right)=P\left({U}_{ik}-{U}_{ij}\le 0, \forall k\ne j\right)$$

3 $$=P({\varepsilon }_{ik}-{\varepsilon }_{ij}\le {x}_{i}{\prime}{\beta }_{j}-{x}_{i}{\prime}{\beta }_{k}, \forall k\ne j)$$

Assuming that{${\varepsilon }_{ij}$ is iid and obeys the gumbel distribution, then formula (3) can be written as:

4 $$P\left({Option}_{ij}=j|{x}_{i}\right)=\frac{\mathrm{exp}({x}_{i}{\prime}{\beta }_{j})}{\sum_{k=1}^{J}\mathrm{exp}({x}_{i}{\prime}{\beta }_{k})}.$$

Obviously, the sum of the probabilities of choosing the three types of industrial organization models is 1, that is $\sum_{k=1}^{J}P\left({Option}_{ij}=j|{x}_{i}\right)=1$. To estimate the formula (4), this paper takes the wheat growers who have not yet participated in any industrial organization model, namely the "smallholder farmer" industrial organization model, as the reference group. β_j_ is the estimated parameter.

#### Test model for the effect of industrial organization model on wheat crop yields

To investigate the impact of wheat growers’ participation in different modes of industrial organization on their crop yields, this paper constructs the following model:

5 $$In{c}_{i}={\gamma }_{0}+{\gamma }_{1}{Option}_{i}+{Z}_{i}{\prime}\rho +{\varepsilon }_{i},$$

6 $$In{c}_{i}={\gamma }_{0}+{\gamma }_{1}{Option}_{ij}+{Z}_{i}{\prime}\rho +{\varepsilon }_{i}.$$

Among them, $In{c}_{i}$ represents the wheat planting income of wheat grower *i*, which is expressed by the average planting income per mu. ${Option}_{i}$ indicates that the industrial organization model of wheat growers *i* is two-dimensional choice decision, and its setting is same with formula (1). In addition, to further investigate the impact of different industrial organization models on the planting income of wheat growers, this paper replaces ${Option}_{i}$ in formula (5) with ${Option}_{ij}$, where *j* represents the type of industrial organization model. This paper including smallholder farmer, farmers + cooperatives/agricultural enterprises, farmers + village collectives + cooperatives/agricultural enterprises. In order to eliminate the problem of multicollinearity, formula (6) takes smallholder farmer as the benchmark. γ_0_ is the intercept, and γ_1_ is the estimated parameter. If it is significantly positive, it shows that the participation of wheat growers in agricultural industrial organizations can significantly improve their planting income. ε_i_ is a random interference.

To further reveal the influence mechanism of different industrial organization modes on the planting income of wheat growers, this paper uses the Materialized cost per mu of wheat planting (seeds, fertilizers, pesticides) (*Cost*_*materialized*_i_), machine cost per mu (*cost*_*machine*_i_), yield per mu (*yield*_i_) and wheat sales price (*price*_i_) replace the explanatory variables in formulas (5) and (6), and re-model regression.

## Empirical results analysis

### Industrial organization choice decision of wheat growers

Table 5 reports the estimation results for both the Logit model and the Linear Probability Model (LPM) in columns (1) and (2) respectively. The statistics at the bottom of columns (1) and (2) indicate good model fit for both. Examining the estimated coefficients, it becomes evident that variables such as the educational level of the household head, training experience, wheat grower's land quality, operational scale, and family labor endowment significantly influence the decision-making of wheat growers regarding their choice of industrial organization model. As this paper concerns wheat cultivation in China, based on the national context of China and the practical situation of the surveyed questionnaire subjects, the data units in this paper are in "亩" (mu) (1 mu = 0.06 hectares).

**Table 5:** Decision-making of wheat growers participating in industrial organization model.

Variable	Logit	LPM	Multinomial logit model [control group: small farmers]	
	(1)	(2)	(3) Farmers + cooperatives/enterprises	(4) Farmers + village collectives + cooperatives/enterprises
Age of householder	0.007 (0.006)	0.0013 (0.0013)	0.003 (0.007)	0.016** (0.008)
Gender of householder	0.084 (0.188)	0.019 (0.042)	0.0245 (0.206)	0.199 (0.253)
Education level of householder	0.232** (0.108)	0.057** (0.024)	0.179* (0.119)	0.327** (0.142)
Householder training experience	0.971*** (0.212)	0.239*** (0.046)	0.684*** (0.226)	1.928*** (0.470)
Cadre family marriage	0.051 (0.114)	− 0.003 (0.025)	0.047 (0.126)	0.055 (0.146)
Plain	0.147 (0.123)	0.03 (0.027)	0.063 (0.134)	0.301* (0.166)
Farmland confirmation	− 0.208 (0.170)	− 0.047 (0.037)	− 0.187 (0.184)	− 0.254 (0.222)
Cultivated land quality	0.331*** (0.115)	0.07*** (0.025)	0.269** (0.127)	0.431*** (0.145)
Farmland transferred in	0.713*** (0.125)	0.187*** (0.027)	0.846*** (0.142)	0.497** (0.161)
Size of family members	− 0.016 (0.028)	− 0.002 (0.006)	− 0.024 (0.031)	− 0.003 (0.035)
The scale of wheat operation	0.001*** (0.0002)	0.0002*** (0.00003)	0.0012*** (0.0002)	0.0014*** (0.0002)
Proportion of migrant workers	− 0.001 (0.003)	− 0.0006 (0.0006)	− 0.004 (0.003)	0.0024 (0.004)
Labour share	− 0.005* (0.003)	− 0.001* (0.0006)	− 0.006** (0.003)	− 0.004 (0.004)
Irrigation conditions	0.0004 (0.002)	0.0003 (0.0003)	− 0.001 (0.002)	0.0037** (0.002)
Intercept item	− 1.660*** (0.458)	0.139 (0.101)	− 1.310*** (0.500)	− 4.643*** (0.716)
Sample size	1870	1870	1870	
Pseudo R2/R2	0.073	0.081	0.058	
LR chi2/F value	184.42***	11.73***	225.9***	

If P < 0.05, the significance is * ; If P < 0.01, the significance is **; If P < 0.001, the significance is ***.

Specifically, if the householder has a high degree of education or training experience, the probability of wheat growers participating in the agricultural industry organization mode is high. The results of the linear probability model show that participating in the industrial organization model will increase by 5.9% if the householder has a high school education or higher, and participating in the agricultural industrial organization model will increase by 24.1% if the householder has training experience. The reason could be that householders with higher education and technical training have a better understanding of the welfare improvements brought by participating in the industrial organization model, and agricultural enterprises or farmers cooperatives are more likely to absorb householders with higher education and technical training to participate in agricultural industrial organizations. The higher cultivated land quality of wheat growers, the higher the possibility of participating in the industrial organization model. The larger the scale of wheat growers and cultivated land transfer, the more likely they are to participate in the industrial organization model. This conclusion is consistent with the findings^11–13^. The possible reason is that farmers' demand for stable sales increases with the expansion of the planting scale. However, adopting modern industrial organization model can promote a more stable trading relationship between farmers and modern industrial organizations. Moreover, to save fixed transaction costs, agricultural enterprises and farmers cooperatives are more inclined to sign contracts with large-scale farmers^14^. The richer the labour endowment of wheat growers, the more likely they are to participate in the industrial organization model. The reason could be that the more labour-rich growers, the less dependent on agriculture, and the lower their willingness to participate in industrial organization models.

Columns (3) and (4) in Table 5 report the fitting results of the multinomial Logit model. It is show that the higher the education level of the householder, the training experience, the cultivated land quality of the wheat growers, and the scale of operation significantly affect the decision-making of the industrial organization model of the wheat growers. The difference is that the higher the age of the householder, the more likely it is to participate in the organizational model of "household + village collectives + farmers cooperatives/agricultural enterprises", which is not significant in sequence (3). The reason could be that farmers cooperatives or enterprises do not favor older farmers. On the contrary, village collectives show enough inclusiveness to older farmers. In plain areas or areas with better irrigation conditions, wheat growers are more likely to participate in the organizational model of "household + village collectives + farmer cooperatives/agricultural enterprises". The reason may be that the village collectives in the region are more inclined to intervene in the change of industrial organization mode.

### The impact of farmers' participation in industrial organization on wheat planting income

Wheat growers participating in industrial organizations and those not yet participating in any industrial organization are not randomly selected. Table 5 shows that wheat growers with higher education, training experience, transfer to farmland, better-cultivated land quality and larger planting scale are more likely to participate in industrial organization. Therefore, if the OLS estimation is directly performed on formulas (5) and (6), it is likely to lead to the deviation of the estimation results because of ignoring the sample selection problem. The propensity score matching (PSM) method proposed by Rosenbaum et al. has been widely used to alleviate the sample selection problem^15^. However, PSM requires all matched covariates to pass the balance test, but in practice, it is difficult for multidimensional covariates to pass the balance test consistently, and the resulting error level is often higher than 5%^16^. Entropy Balancing (EB) proposed by Hainmueller is a method for re-weighting multi-dimensional variables. The principle is to set certain moment constraints on covariates that may cause bias, so that the treatment and the control groups are balanced under constraints to obtain the weight of each sample. The larger the weight is, the closer the characteristics of the control group samples are to the treatment group, and the control group reweighted by entropy balance is closer to the treatment group9^17^. Therefore, after data preprocessing using entropy balance, this paper uses the OLS regression model to formulas (5) and (6).

The results of Table 6 show that before the entropy balance treatment, the mean value, standard deviation and skewness of the householder, family and village characteristics are significantly different between the treatment and control groups. After the entropy balance treatment, the mean value, standard deviation and skewness of the treatment and control groups' characteristic variables were close.

**Table 6:** Entropy balance processing results.

Variable	Treat			Control		
	Mean	Variance	Skewness	Mean	Variance	Skewness
Before						
Age of householder	46.99	74.8	− 0.3425	47.22	85.74	− 0.2196
Gender of householder	0.9283	0.0666	− 3.319	0.914	0.0786	− 2.954
Education level of householder	0.6605	0.2244	− 0.6781	0.5891	2424	− 0.3623
Householder training experience	0.9633	0.0354	− 4.925	0.8862	0.101	− 2.433
Cadre family marriage	0.301	0.2106	0.8679	0.3047	0.2121	0.8487
Plain	0.7979	0.1614	− 1.484	0.7775	0.1732	− 1.334
Farmland confirmation	0.8994	0.0905	− 2.655	0.9064	0.0849	− 2.791
Cultivated land quality	0.2992	0.2099	0.877	0.2579	0.1916	1.107
Farmland transferred in	0.846	0.1304	− 1.917	0.6877	0.215	− 0.8102
Size of family members	4.964	4.103	6.872	4.938	3.63	4.639 7.408
The scale of wheat operation	406.4	446,503	7.716	201.1	120,117	− 1.139
Proportion of migrant workers	13.64	363.3	1.44	16.92	397.4	− 0.2196
Labour share	61.15	424.4	0.252	62.27	423.5	− 2.954
Irrigation conditions	75.2	1035	− 1.085	75.42	1111	− 0.3623
After						
Age of householder	46.99	74.8	− 0.3425	47	74.82	− 0.3434
Gender of householder	0.9283	0.0666	− 3.319	0.9283	0.0666	− 3.32
Education level of householder	0. 6605	0.2244	− 0.6781	0.6607	0.2245	− 0.6787
Householder training experience	0.9633	0.0354	− 4.925	0.9633	0.0354	− 4.925
Cadre family marriage	0.301	0.2106	0.8679	0.3009	0.2106	0.8683
Plain	0.7979	0.1614	− 1.484	0.798	0.1614	− 1.484
Farmland confirmation	0.8994	0.0905	− 2.655	0.8994	0.0905	− 2.656
Cultivated land quality	0.2992	0.2099	0.877	0.2991	0.2099	0.8775
Farmland transferred in	0.846	0.1304	− 1.917	0.8461	0.1304	− 1.918
Size of family members	4.964	4.103	6.872	4.964	4.102	6.871
The scale of wheat operation	406.4	446,503	7.716	406.4	446,295	7.718
Proportion of migrant workers	13.64	363.3	1.44	13.64	363.3	1.439
Labour share	61.15	424.4	0.252	61.15	424.4	0.2517
Irrigation conditions	75.2	1035	− 1.085	75.2	1035	− 1.085

Previous studies have shown that compared with traditional industrial organization models, modern industrial organization models such as "household + cooperative" and "household + enterprise" have increased farmers' income through production services, premium payment and financial support. According to Chen Chao et al. when compared to the traditional market trading model, modern industrial organization models such as "household + farmer cooperatives" and "household + agricultural enterprises" can change the traditional extensive production mode of smallholder farmer to a certain extent, optimize the allocation of farmers' production factor, and then guide farmers to carry out scientific and standardized production^18^. To validate the impact of wheat growers' participation in industrial organizations on their planting benefits, this paper uses the entropy balancing data preprocessing method to process household characteristics, family features, and village characteristics. Then, it conducts OLS estimation on Eqs. (5) and (6). The empirical results, with some missing data excluded, are presented in Table 7. In Table 7, columns (1) and (3) show that without entropy balancing treatment for the treatment and control groups, the effect of wheat farmers&apos; participation in agricultural industry organizations on their planting income is not significant. Conversely, column (2) indicates that after undergoing entropy balancing treatment, the "participation in industry organization" variable is significantly positive with an estimated coefficient of 0.0183. This suggests that under the premise of controlling sample selection bias, wheat farmers participating in any agricultural industry organization model can significantly increase their net planting income (higher yield, reduced costs), approximately by 18.3 CNY per mu. The results in column (4) demonstrate that compared to small-scale farmers with dispersed operations, wheat farmers participating in the "household + farmer cooperative/agricultural enterprise" planting model can significantly increase their net planting income by approximately 20.6 CNY per mu. Additionally, wheat farmers participating in the "household + village collective + farmer cooperative/agricultural enterprise" planting model can increase their planting income by approximately 14.1 CNY per mu.

**Table 7:** The impact of participating in industrial organization on wheat crop yields.

Variable	(1) Without entropy balance treatment	(2) After entropy balance treatment	(3) without entropy balance treatment	(4) After entropy balance treatment
Participation in industrial organization	0.00313 (0.00528)	0.0183*** (0.00597)		
Small farmers + farmer cooperatives/agricultural enterprises			0.00576 (0.00580)	0.0206*** (0.00676)
Farmers + village collectives + cooperatives/enterprises			− 0.00174 (0.00690)	0.0141** (0.00675)
Control variables	Yes	Yes	Yes	Yes
_cons	0.150*** (0.0232)	0.108*** (0.0325)	0.148*** (0.0232)	0.106*** (0.0326)
N	1832	1832	1832	1832
R^2^	0.049	0.062	0.049	0.062

If P < 0.05, the significance is * ; If P < 0.01, the significance is **; If P < 0.001, the significance is ***.

### Analysis of the influence mechanism of participating in industrial organization on farmers' crop yields

To further reveal the influence mechanism of agricultural industrial organization mode on the wheat growers' crop yields, this paper uses the explanatory variables of materialized cost, machinery cost, average yield per mu and wheat sales price replacement formulas (5) and (6), and re-model regression. The results of Table 8 show that compared with wheat growers who have not yet participated in any industrial organization, participating in the industrial organization has significantly increased the average yield per mu and sales price, the average yield per mu has increased by 31.15 kg, and the sales price has increased by 0.0262 CNY CNY per kg. The impact of participating in the agricultural industrial organization model on the mechanical cost input and materialized cost input of wheat growers is not significant. In other words, the impact of the agricultural industry organization model on wheat growers is mainly achieved by increasing their land output rate and market bargaining power.

**Table 8:** The influence mechanism of participating in industrial organization on the wheat growers' crop yield.

Variable	(1) Machine cost	(2) Materialization cost	(3) Average yield per mu (500 g)	(4) Sales price (CNY/500 g)
Participation in industrial organization	6.503 (7.429)	− 0.0105 (0.221)	37.04 (27.15)	0.0250* (0.0144)
Control variables	Yes	Yes	Yes	Yes
_cons	60.68** (32.4)	3.988*** (1.204)	735.1*** (125.3)	1.455*** (0.621)
N	1774	1733	1851	1797
R-sq	0.038	0.049	0.140	0.055

If P < 0.05, the significance is * ; If P < 0.01, the significance is **; If P < 0.001, the significance is ***.

Table 9 reports the estimated results which indicate that the findings indicate that participation in the "household + farmer cooperatives/agricultural enterprises" model can significantly improve wheat growers' land productivity and market bargaining power. Compared with the decentralized management mode of smallholder farmer, wheat growers participating in the "smallholder household + farmers cooperatives/agricultural enterprises" organization mode will increase the average yield per mu by 90.64 kg and the sales price of wheat by 0.0488 CNY/kg. On the contrary, village collective intervention in the industrial organization model only helps to improve the market bargaining power of wheat growers, and the wheat sales price of wheat growers will significantly increase by 0.0588 CNY/kg CNY (Supplementary Information).

**Table 9:** The impact of participating in different industrial organization models on farmers' crop yields.

Variable	(1) Machine cost	(2) Materialization cost	(3) Average yield per mu (500 g)	(4) Sales price (CNY/500 g)
Small farmers + farmers cooperative agriculture/enterprise	9.252 (7.934)	0.0592 (0.227)	47.91* (27.57)	0.0227 (0.0146)
Small farmers + village collective + farmer cooperatives/agricultural enterprises	1.586 (9.662)	− 0.134 (0.265)	17.71 (31.43)	0.0292* (0.0164)
Control variables	Yes	Yes	Yes	Yes
_cons	58.54** (32.45)	3.927*** (1.218)	726.6*** (126.1)	1.457*** (0.0624)
N	17,874	1733	1821	1797
R-sq	0.039	0.050	0.141	0.088

## Research conclusion

### Research conclusion

The scientific analysis and empirical research on the organizational models of the wheat industry in Anhui Province hold significant practical significance for China’s achievement of high-quality and high-price grain production and the enhancement of food security. In order to conduct a more comprehensive empirical study on the optimal organizational models for wheat production, this paper focused on 2032 wheat growers in Anhui Province. It employed Logit models, multinomial Logit models, and entropy balance-OLS regression methods, selecting factors such as wheat planting income, costs, and farmer endowments as indicators. The study examined the influencing factors of wheat farmers' participation in agricultural industry organization models and the impact of their participation decisions on planting income. The results indicate that:

1. The richer the resource endowment of wheat growers, the higher the probability of their participation in the agricultural industrial organization model. The householder's education level, training experience, family-cultivated land quality, operation scale and labour endowment are essential factors influencing the wheat growers' participation in the decision-making of industrial organization mode. As the household head's training experience becomes more extensive, the probability of their participation in agricultural industry organization models will increase by 23.9%. Similarly, as the educational level of the household head rises, the likelihood of their participation in industry organization models will increase by 5.7%. The policy implication is that priority should be given to fostering the development of agro-industrial organizations in regions with rich resource endowments of farm households.
2. The participation of wheat growers in agricultural industrial organizations can significantly improve their income. Compared with the participation of "household + farmers cooperatives/agricultural enterprises" mode, the effect of industrial organization income increase is more obvious. This significantly increases their net planting income by approximately 20.6 CNY per mu. Its policy implication is that the government should to actively guide farmers to participate in agricultural industrial organizations, primarily based on the "household + farmers cooperatives/agricultural enterprises" model, which is an effective measure to increase farmers' income.
3. There are variations in the impact mechanisms on planting income for wheat farmers participating in different agricultural industry organization models. Wheat farmers engaged in the "household + farmer cooperative/agricultural enterprise" model of industry organization will see an increase of 28.9 kg in yield per mu, and the selling price of their wheat will rise by 0.0584 CNY per kilogram. Therefore, the income enhancement effect of the "household + farmer cooperative/agricultural enterprise" model primarily stems from improved land productivity and market bargaining power. On the other hand, for those participating in the "household + village collective + farmer cooperative/agricultural enterprise" model, the selling price of wheat will significantly increase by 0.0588 CNY per kilogram. Hence, the income enhancement effect of the "household + village collective + farmer cooperative/agricultural enterprise" model primarily arises from the improvement in market bargaining power. The policy implication is that priority should be given to the development of the "household + farmer cooperative/agricultural enterprise" model of industry organization. Meanwhile, the development of the "household + village collective + farmer cooperative/agricultural enterprise" model can be considered on a case-by-case basis, as it serves as a beneficial complement to the former.

### Policy suggestion

Based on the above conclusions, the following recommendations are proposed: (1) Actively encourage farmers to participate in crop industry organizations. Governments at all levels should intensify efforts to promote crop industry organizations, guiding farmers to understand the operational models of these organizations. Tailor guidance to meet the diverse needs of farmers and encourage their participation in various forms of crop industry organizations according to local conditions. (2) Support the development of crop industry organizations. The primary reason for farmers not participating in crop industry organizations is the limited availability of such models. Relevant authorities should provide support for the development of crop industry organizations through financial assistance, favorable policies, and incentives. Encourage leading enterprises to establish industrial bases and initiate high-quality cooperatives. Motivate village collectives to actively engage in the operation of these organizations, continuously enhancing the involvement of key players in the industry chain. This should lead to a shift from binary cooperation to diversified collaboration. (3) Strengthen the management of crop industry organizations. Emphasize comprehensive supply chain management, enhance processing capabilities, and improve the profitability of these organizations. Establish robust trust mechanisms within these organizations and expedite the implementation of organization branding development strategies.

### Supplementary Information

Supplementary Information 1.

Supplementary Information 2.

Supplementary Information 3.
