# Supplementary material for: The impact of participation in agricultural industry organizational models on crop yields: evidence from Chinese wheat growers
Source: Sci Rep. 2023 Oct 18;13:17779. doi: 10.1038/s41598-023-43879-0 (PMC10584860; doi:10.1038/s41598-023-43879-0)
Supplement: Supplementary file 3 — Supplementary Information 3. [file 41598_2023_43879_MOESM3_ESM.docx]

Statement

The file named "Raw Data" contains all the data in STATA format for the article, while the file named "DO" contains the running instructions in STATA format. Please review them.
